# Supplementary material for: Targeting HIF-2α in colorectal cancer reveals a cholesterol biosynthesis–dependent ferroptotic vulnerability
Source: Cancer Metab. 2026 Feb 12;14:5. doi: 10.1186/s40170-026-00421-w (PMC12998000; doi:10.1186/s40170-026-00421-w)
Supplement: Supplementary file 1 — Supplementary Material 1 [file 40170_2026_421_MOESM1_ESM.pptx]

## Slide 1
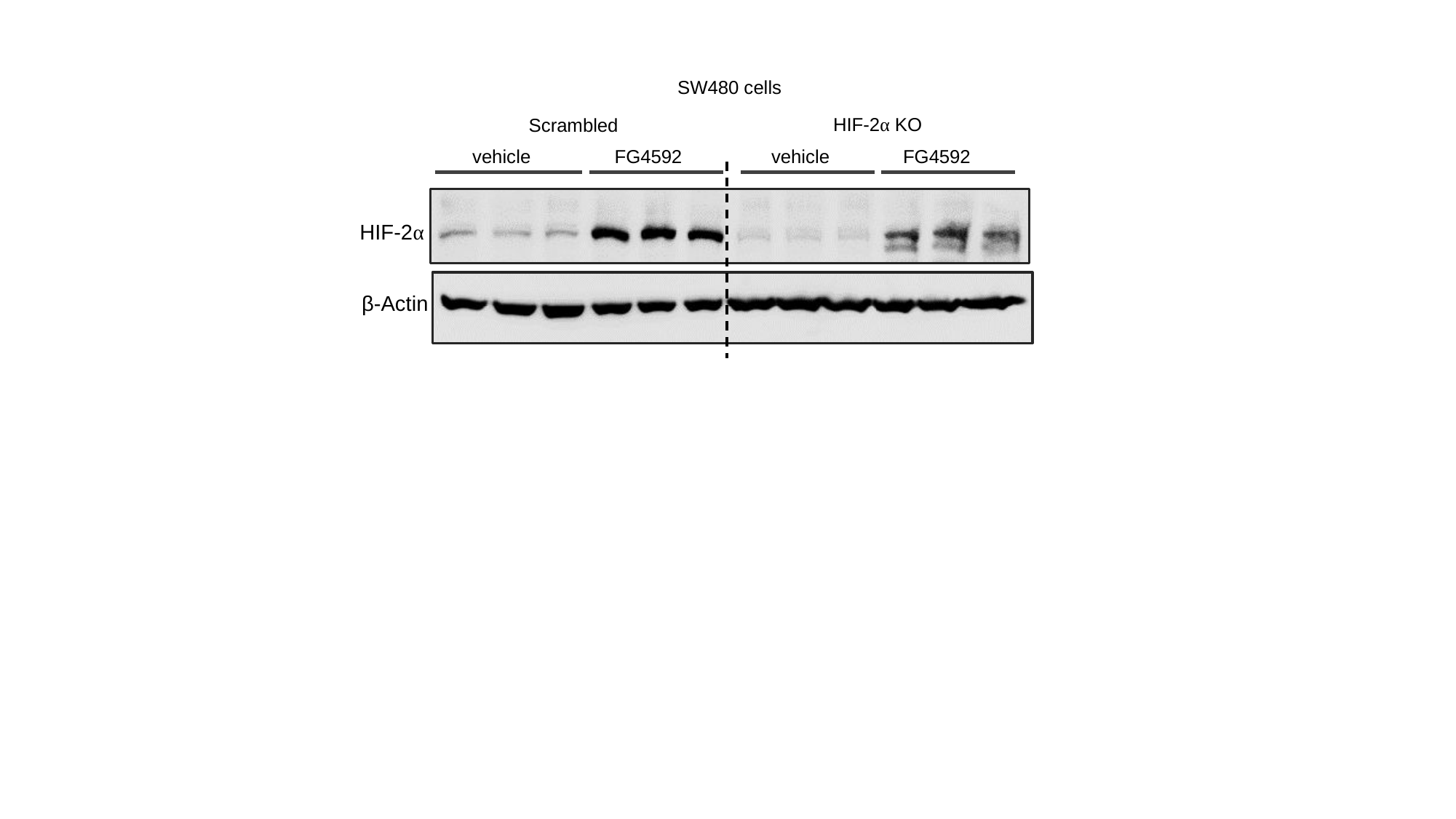

SW480 cells
 Scrambled
 vehicle FG4592 vehicle FG4592
 HIF-2α KO
HIF-2α
β-Actin

## Slide 2
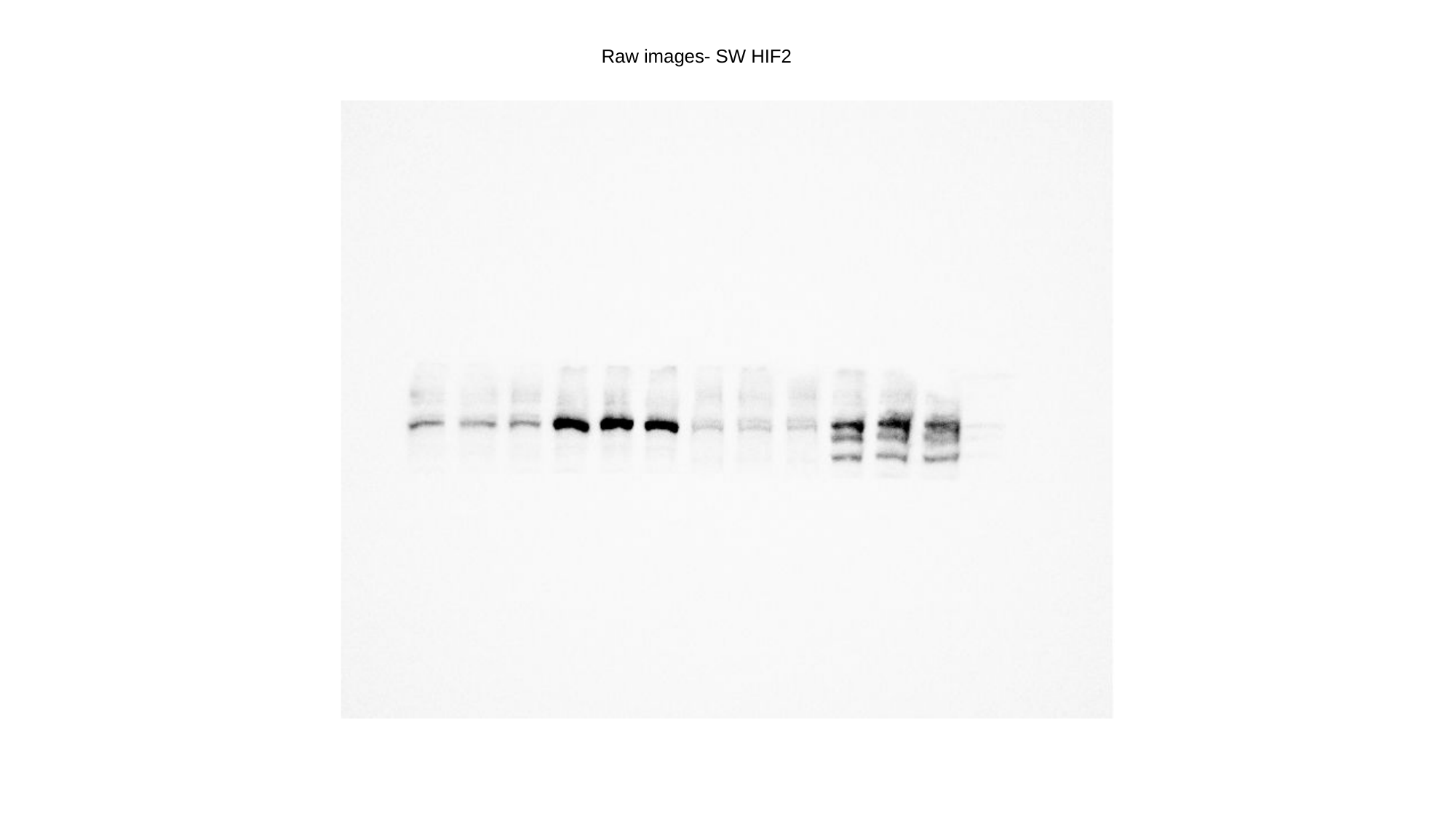

Raw images- SW HIF2

## Slide 3
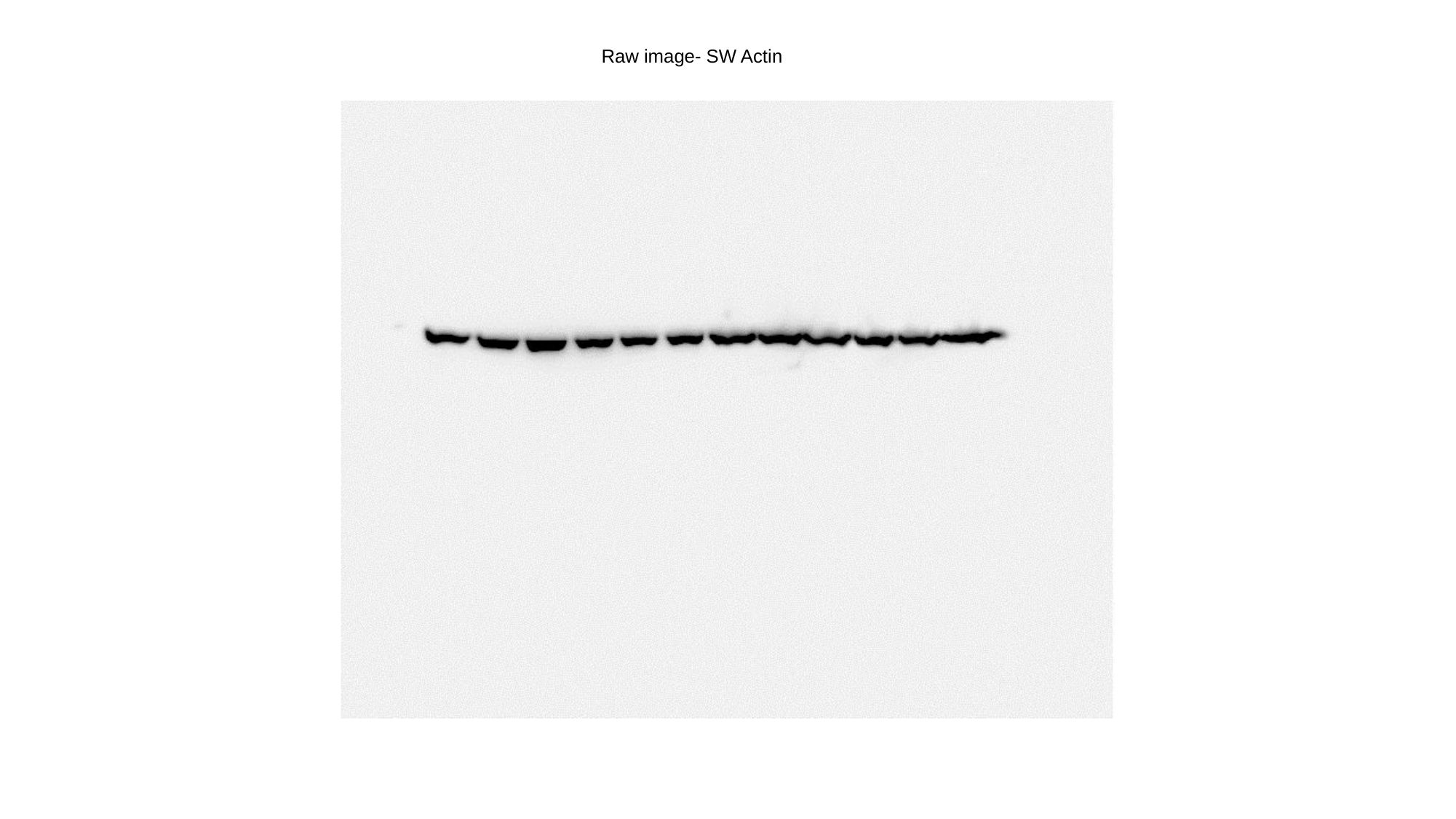

Raw image- SW Actin
