# Supplementary material for: Targeting HIF-2α in colorectal cancer reveals a cholesterol biosynthesis–dependent ferroptotic vulnerability
Source: Cancer Metab. 2026 Feb 12;14:5. doi: 10.1186/s40170-026-00421-w (PMC12998000; doi:10.1186/s40170-026-00421-w)
Supplement: Supplementary file 2 — Supplementary Material 2 [file 40170_2026_421_MOESM2_ESM.docx]

**Supplemental Figure 1.**

**
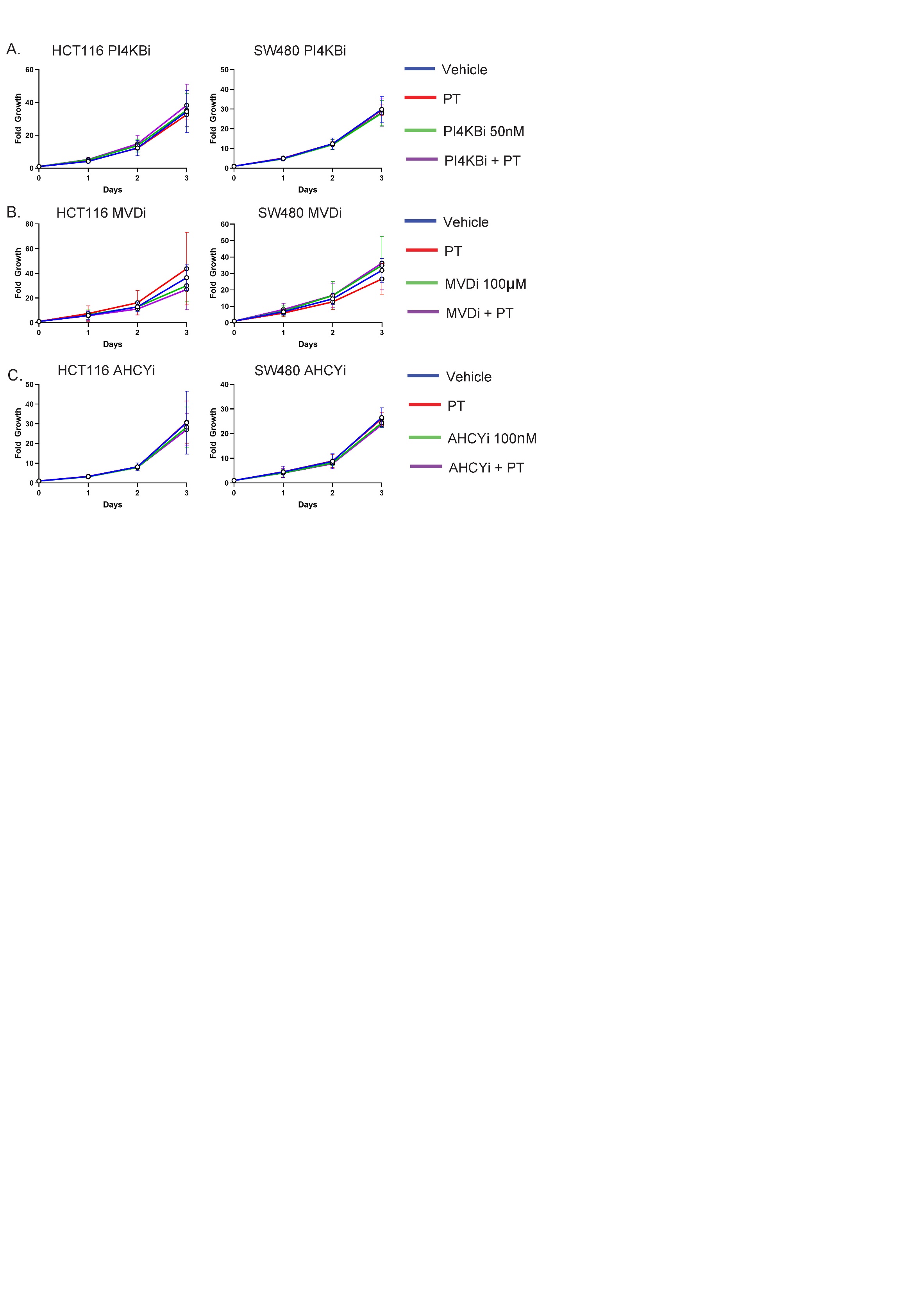
**

**Supplemental Figure 1. Metabolic vulnerabilities in PI4K, MVD, and AHCY do not sensitize CRC cells to HIF2α inhibition. (A)** Proliferation of HCT116 and SW480 cells treated with PI4KB inhibitor IN-10, either alone or in combination with PT2385 (PT). **(B)** Proliferation of HCT116 and SW480 cells treated with MVD inhibitor 6-fluoromevalonate (6FM), alone or in combination with PT2385. **(C)** Proliferation of HCT116 and SW480 cells treated with dual EZH2 and AHCY inhibitor, 3-deazaneplanocin A hydrochloride (DZNep). Data are presented as mean ± SEM. Representative graphs shown. Proliferation data performed in triplicates and repeated 1-3 times.


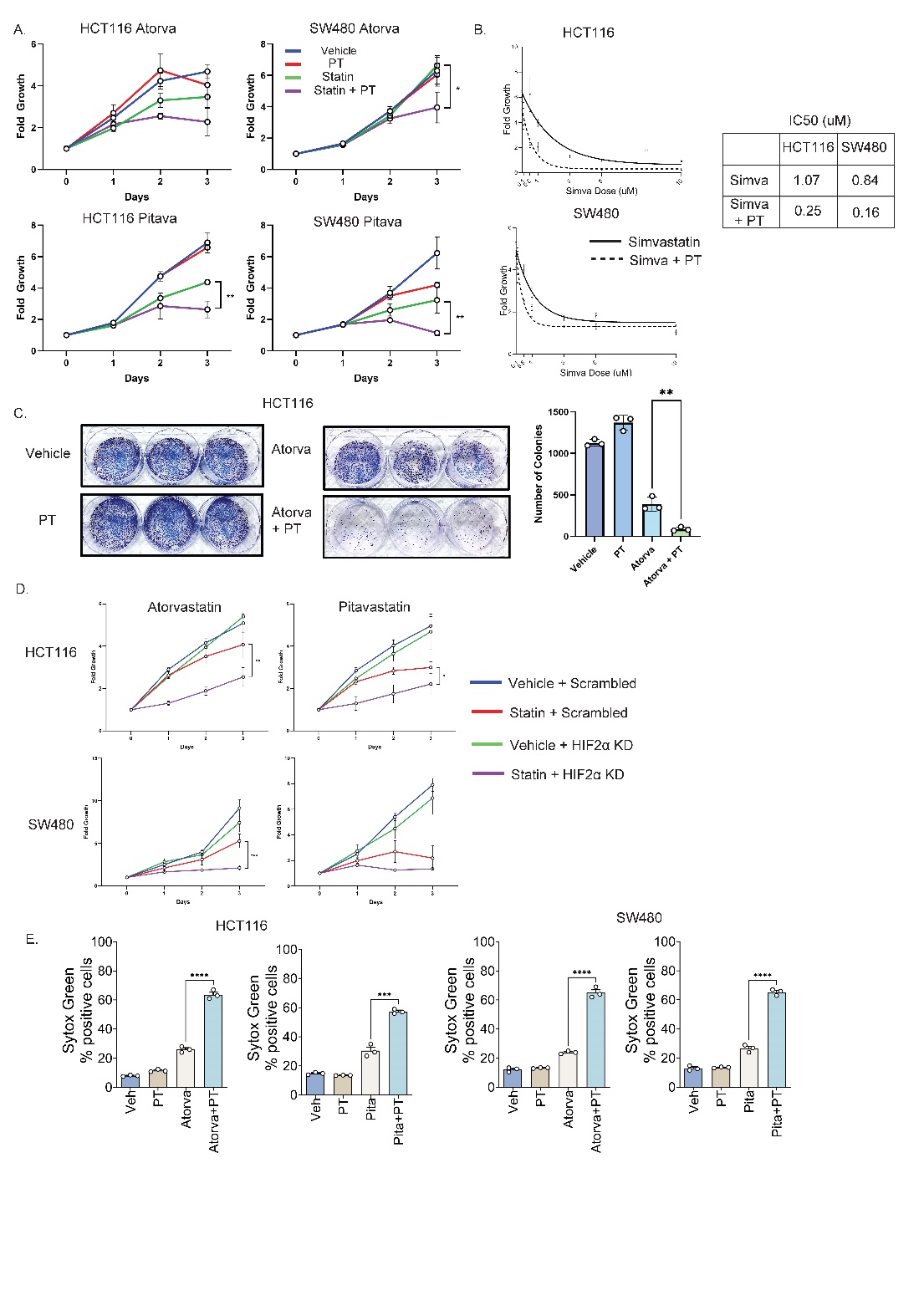
**Supplemental Figure 2.**

**Supplemental Figure 2. Atorvastatin and pitavastatin demonstrate similar effects in suppressing CRC and inducing cell death as simvastatin when combined with HIF-2α inhibition. (A)** Proliferation assays in HCT116 and SW480 cells treated with atorvastatin (atorva) and pitavastatin (pitava) either alone or in combination with PT2385 (PT). **(B)** The half maximal inhibitor concentration (IC_50_) of simvastatin is reduced in the presence of PT2385. **(C)** Colony formation assays demonstrate that atorvastatin reduces colony number. This effect is further potentiated with PT2385. **(D)** HCT116 and SW480 cells transduced with shRNA targeting HIF2α display decreased proliferation when treated with atorvastatin or pitavastatin. **(E)** Sytox Green assays quantifying cell death in HCT116 and SW280 cells treated with atorvastatin or pitavastatin alone or in combination with PT2385 at 24 hours. Data are presented as mean ± SEM; *p<0.05, **p<0.01, ***p<0.001. Representative graphs shown. Proliferation data performed in triplicates and repeated 1-2 times. Half maximal inhibitor concentration performed in triplicates. Colony formation assay performed in triplicates. Cell death assays performed in triplicates.


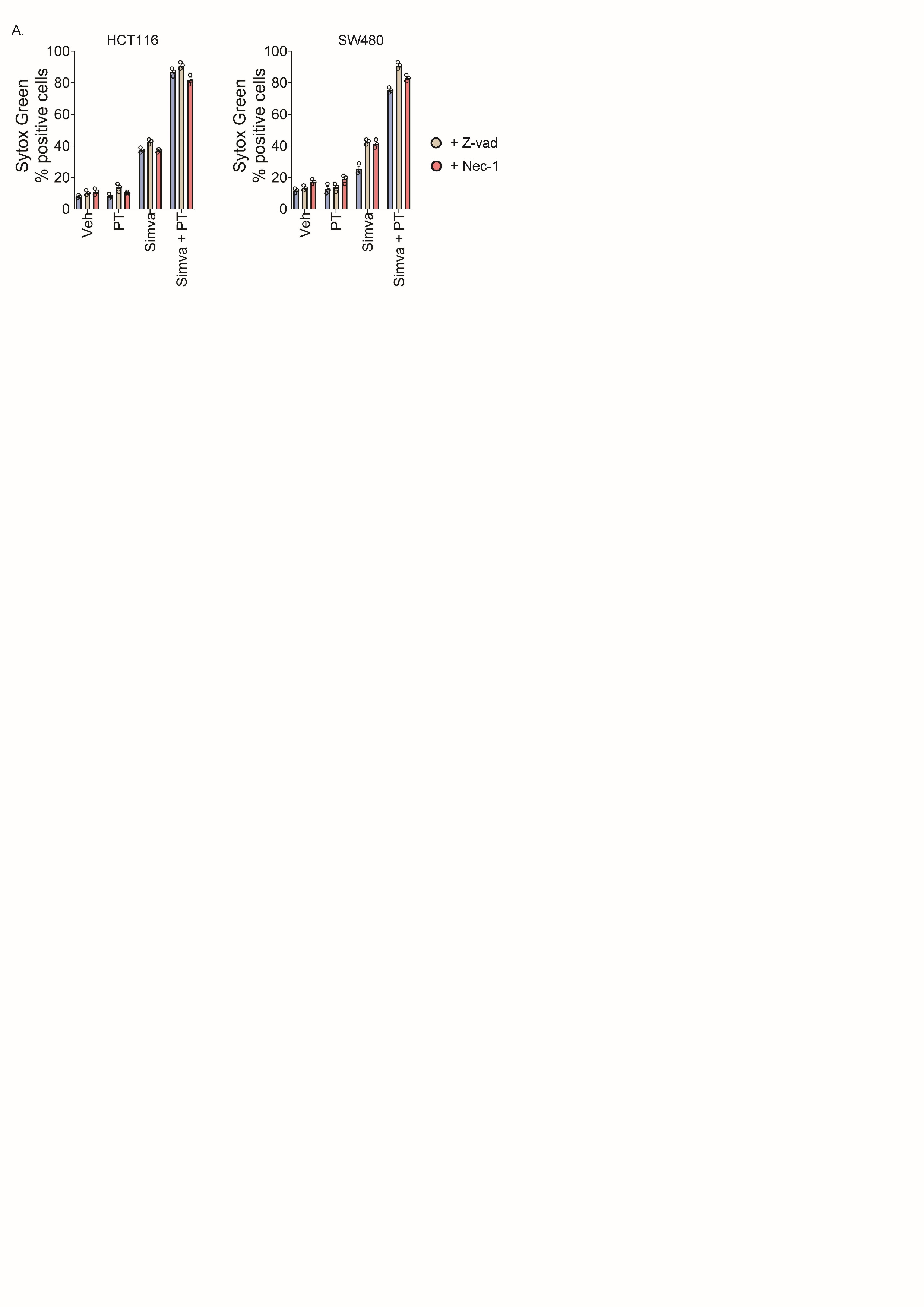
**Supplemental Figure 3.**

**Supplemental Figure 3. Inhibiting apoptosis and necroptosis cell death pathways do not reverse cell death mediated by combination simvastatin and HIF-2α inhibition. (A**) Combined simvastatin (simva) and PT2385 (PT) treatment increases cell death as measured by Sytox Green. This effect is not reversible by the addition of a pan-caspase inhibitor blocking apoptosis-mediated cell death (Z-vad, 10 µM), or a RIP1 inhibitor blocking necroptosis-mediated cell death (Necrostatin-1, Nec-1, 10µM). Data are presented as mean ± SEM. Representative graphs shown. Cell death assays performed in triplicates and repeated two times.

**Supplemental Figure 4.**

**
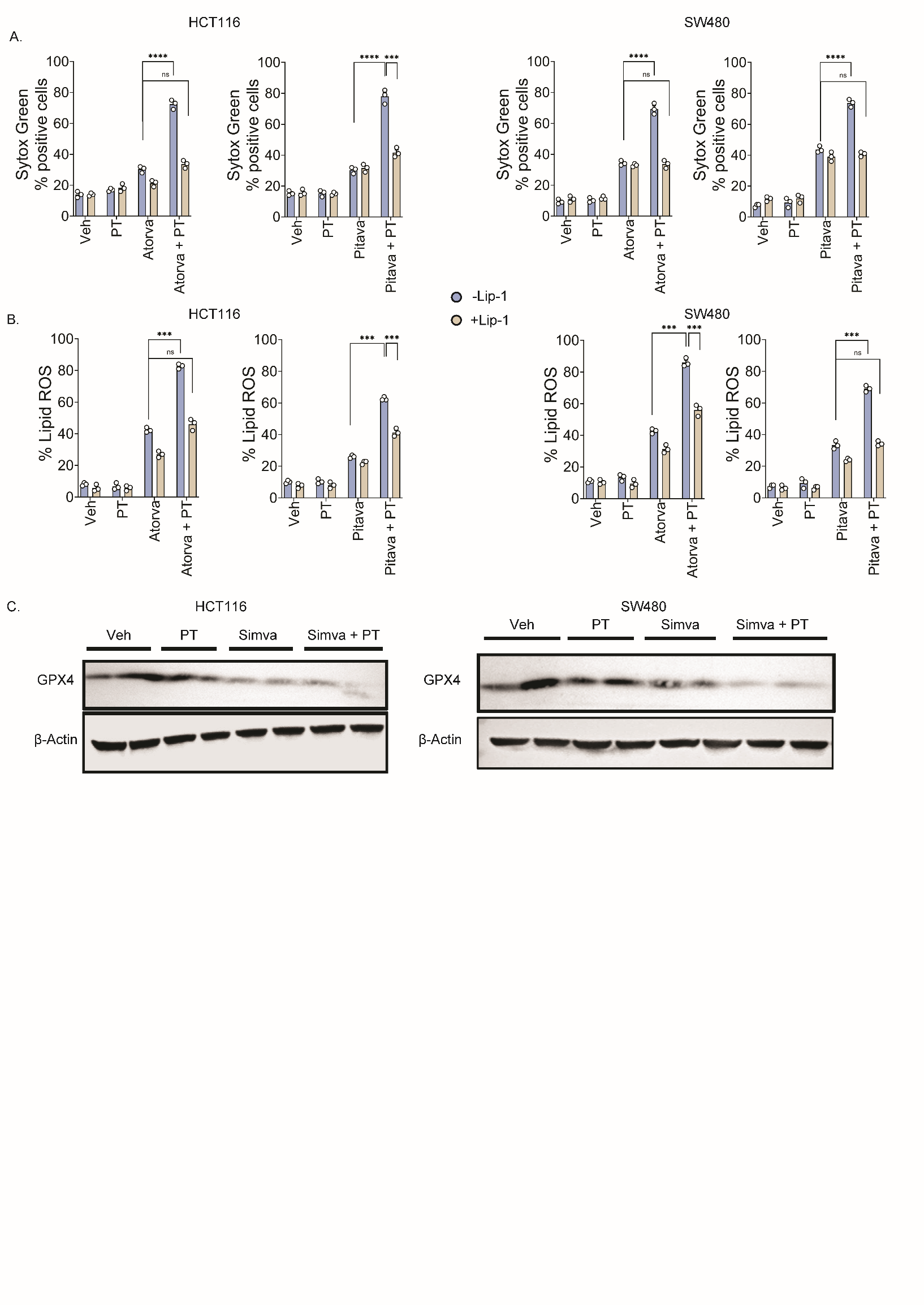
**

**Supplemental Figure 4. Increased cell death and lipid ROS generation caused by atorvastatin or pitavastatin in combination with HIF-2α inhibition is reversible with ferroptosis inhibitor liproxstatin-1. (A**) Combined atorvastatin (atorva) or pitavastatin (pitava) and PT2385 (PT) treatment significantly increases cell death as measured by Sytox Green. This effect is reversible with the addition of ferroptosis inhibitor liproxstatin-1, 1µM (Lip-1). **(B)** Combined atorvastatin or pitavastatin with PT2385 treatment significantly increases lipid ROS. This effect is rescued by the addition of Lip-1. **(C)** Combined simvastatin (Simva) and PT2385 (PT) treatment decreases GPX4 protein levels especially in the SW480 CRC cell line.

Data are presented as mean ± SEM; *p<0.05, **p<0.01, ***p<0.001. Cell death and lipid ROS data performed in triplicates.

**Supplemental Figure 5.**

**
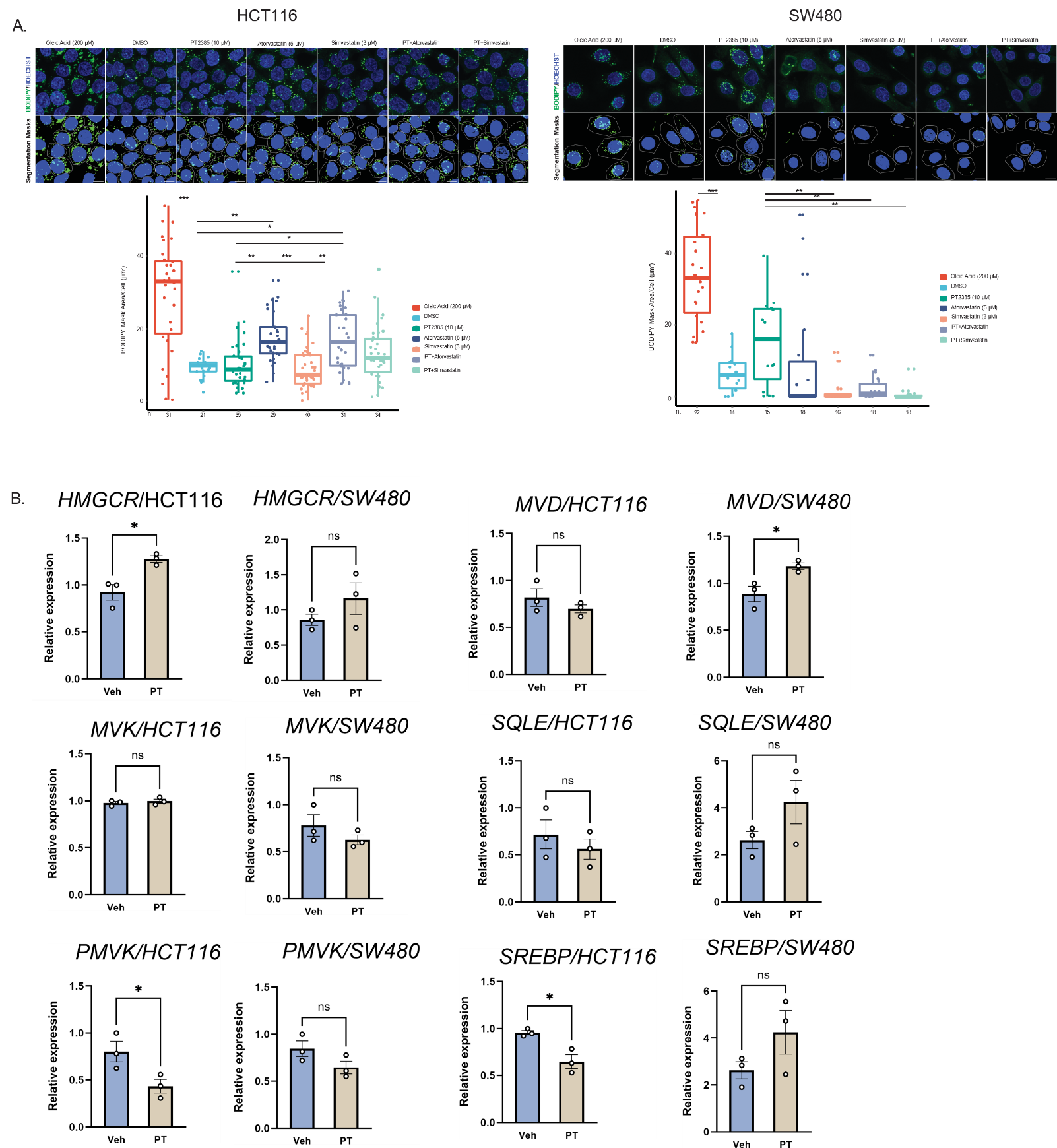
**

**Supplemental Figure 5. Lipid Droplet Quantification. (A**) Lipid droplet area/cell was assessed in HCT116 and SW480 CRC cells using BODIPY 493/503. Cell segmentation and staining area was quantified across oleic acid, DMSO, PT2385, atorvastatin, simvastatin, and combination groups. Oleic acid significantly induces lipid droplet area. PT2385 treatment alone does not significantly alter lipid droplet area. * = p<0.05, ** = p<0.01, and *** = p<0.001. Scale bars = 10 µm **(C)** qRT-PCR Data on PT and cholesterol biosynthesis genes

Data are presented as mean ± SEM; *p<0.05. qRT-PCR performed in triplicates.
